# Visuomotor Entrainment and the Frequency-Dependent Response of Walking Balance to Perturbations

**DOI:** 10.1109/TNSRE.2016.2603340

**Published:** 2016-08-26

**Authors:** Jason R. Franz, Carrie A. Francis, Matthew S. Allen, Darryl G. Thelen

**Affiliations:** Joint Department of Biomedical Engineering, University of North Carolina at Chapel Hill and North Carolina State University, Chapel Hill, NC 27599 USA; Department of Biomedical Engineering, University of Wisconsin-Madison, Madison, WI USA 53706; Departments of Engineering Physics and Mechanical Engineering, University of Wisconsin-Madison, Madison, WI 53706 USA; Departments of Mechanical and Biomedical Engineering and Orthopedics and Rehabilitation, University of Wisconsin-Madison, Madison, WI 53706 USA

**Keywords:** Sensorimotor, Stability, Virtual Reality, Posture, Gait

## Abstract

Visuomotor entrainment, or the synchronization of motor responses to visual stimuli, is a naturally emergent phenomenon in human standing. Our purpose was to investigate the prevalence and resolution of visuomotor entrainment in walking and the frequency-dependent response of walking balance to perturbations. We used a virtual reality environment to manipulate optical flow in ten healthy young adults during treadmill walking. A motion capture system recorded trunk, sacrum, and heel marker trajectories during a series of 3-min conditions in which we perturbed a virtual hallway mediolaterally with systematic changes in the driving frequencies of perceived motion. We quantified visuomotor entrainment using spectral analyses and changes in balance control using trunk sway, gait variability, and detrended fluctuation analyses (DFA). ML kinematics were highly sensitive to visual perturbations, and instinctively synchronized (i.e., entrained) to a broad range of driving frequencies of perceived ML motion. However, the influence of visual perturbations on metrics of walking balance was frequency-dependent and governed by their proximity to stride frequency. Specifically, we found that a driving frequency nearest to subjects' average stride frequency uniquely compromised trunk sway, gait variability, and step-to-step correlations. We conclude that visuomotor entrainment is a robust and naturally emerging phenomenon during human walking, involving coordinated and frequency-dependent adjustments in trunk sway and foot placement to maintain balance at the whole-body level. These findings provide mechanistic insight into how the visuomotor control of walking balance is disrupted by visual perturbations and important reference values for the emergence of balance deficits due to age, injury, or disease.

## I. Introduction

The control of balance in walking is highly dependent on the integration of reliable visual, vestibular, and somatosensory feedback [1-5]. A deterioration in the quality of sensory feedback or in its integration may predispose an individual to falling. Accordingly, sensory perturbations are increasingly used to study the emergence of balance deficits due to aging, injury, or disease. For example, consistent with studies on the postural control of standing [6-9], our recent findings suggest that old adults exhibit an enhanced susceptibility to balance deficits elicited by visual perturbations during walking [10, 11]. Inter-individual differences in the sensitivity to visual perturbations may thus facilitate the diagnosis of sensory-induced balance deficits. However, these clinical implications are currently limited by our relatively incomplete mechanistic understanding of how the visuomotor control of walking balance is disrupted by visual perturbations, even in healthy young adults.

Using virtual reality to study standing balance control, Dijkstra et al. [12] found that standing sway exhibits naturally emerging synchronization to sinusoidal visual perturbations across a range of frequencies of perceived motion. We refer to this as visuomotor entrainment, which describes the extent to which motor responses instinctively synchronize to visual stimuli. These authors suggested that in standing balance control, visual feedback is actively utilized to minimize the error between visual kinesthesis (i.e., the visual perception of motion, [13]) and actual motion of the head and trunk, thereby leading to naturally emerging entrainment to perceived motion. The seminal papers on this topic in walking instructed subjects to alter their trunk sway to follow sinusoidal changes in perceived motion and investigated the biomechanical consequences [14-17]. In contrast, we [10] and others [18] have discovered evidence that visual perturbation frequencies may emerge, naturally and without instruction, in the spectrum of mediolateral (ML) motion during walking. Thus, visuomotor entrainment may allude to mechanisms by which visual feedback is utilized in motor planning and execution to control walking balance. However, the prevalence and resolution of visuomotor entrainment in walking across a broad range of driving frequencies of perceived ML motion have yet to be systematically explored.

Visuomotor entrainment may also provide important insight into the biomechanical origins of walking balance deficits in the presence of visual perturbations, particularly regarding coordination between posture (i.e., head and trunk stabilization) and adjustments in foot placement. Differing from the continuous control of posture, presumably the neuromechanical origin of visuomotor entrainment, adjustments in foot placement are believed to occur more discretely on a step to step basis [1, 3, 16]. For example, Rankin et al. [19] revealed that muscular activity adjusted foot placement laterally during each swing phase by an amount proportional to ML translation of the trunk relative to the stance foot. Thus, stride frequency may be an important determinant of the efficacy of foot placement control to respond to postural disturbances elicited by entrainment to visual perturbations. However, to our knowledge, no studies have investigated the potentially frequency-dependent response of walking balance control to visual perturbations.

Therefore, the purpose of this study was to investigate the prevalence and resolution of visuomotor entrainment in human walking and its role in the emergence of altered movement patterns due to visual perturbations. We used a virtual reality environment to perturb optical flow with systematic changes in the driving frequencies of perceived mediolateral motion. First, we hypothesized that ML motion in human walking is highly and acutely sensitive to visual perturbations and exhibits naturally emerging entrainment to a broad range of driving frequencies. Second, we hypothesized that despite this broad sensitivity, balance deficits elicited by visual perturbations are frequency-dependent and governed by their proximity to stride frequency. Here, smaller balance deficits would emerge when perturbations allowed sufficient time for corrective actions (i.e., driving frequency ≪ stride frequency) or were effectively mitigated via low-pass neuromechanical filtering (i.e., driving frequency > stride frequency). Finally, by logical extension, we hypothesized that there would be a frequency band of perceived ML motion for which balance deficits in walking would be most prominent. In this study, balance deficits would be evidenced by larger ML trunk motion, increased gait variability, and altered patterns of step-to-step correlations compared to normal, unperturbed walking.

## II. Methods

Ten healthy and physically active adults participated (4F, mean ± standard deviation, age: 24.3 ± 2.9 years, mass: 70.9 ± 11.7 kg, height: 1.80 ± 0.11 m). Before testing, all subjects provided written informed consent as per the University of Wisconsin Health Sciences Institutional Review Board.

### A. Experimental Procedure

**Fig. 1:**
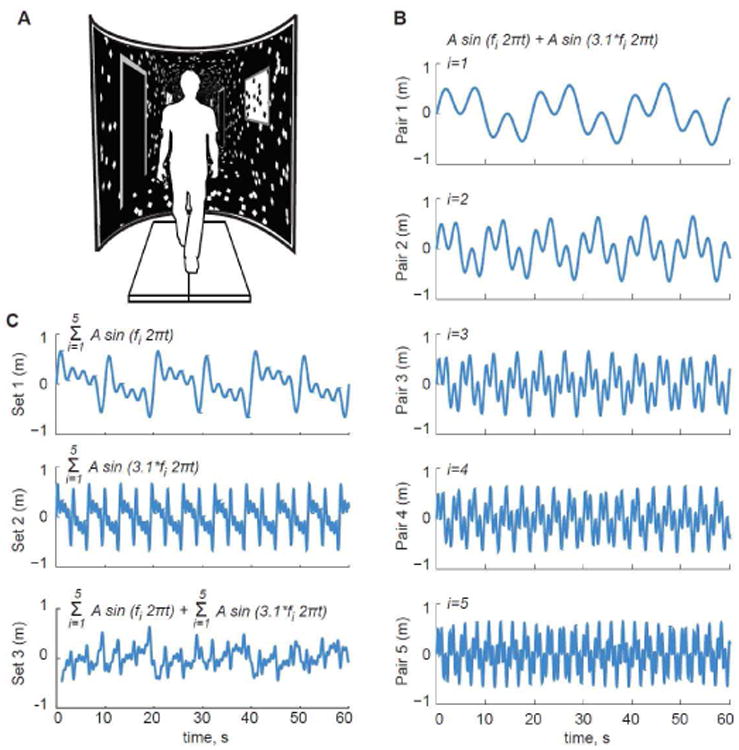
(A) We used a virtual reality environment to perturb optical flow with systematic changes in the driving frequencies of perceived mediolateral motion. In addition to normal, unperturbed walking, our five primary conditions (B) consisted of two sinusoidal driving frequencies each, for which we prescribed 0.326 m amplitudes and f equaled 0.05, 0.10, 0.20, 0.30, and 0.40 Hz (Pair 1-5). (C) Subjects also walked with larger combinations of these driving frequencies. Two of these conditions included 5 driving frequencies with amplitudes of 0.19 m (Set 1: {*f}*, Set 2: 3.1·{*f}*) and one included all 10 driving frequencies with amplitudes of 0.09 m (Set 3).

We recorded subjects' preferred overground walking speed from the average of two times taken to traverse the middle 4 m of a 10 m walkway at a normal, comfortable speed (1.43 ± 0.17 m/s). Subjects then completed all treadmill walking trials on a force-sensing treadmill (Bertec, Corp., Columbus, OH) at their preferred overground speed while watching a speed-matched virtual hallway (Fig. 1A). The virtual hallway was rear-projected onto a semicircular curved screen that surrounded the treadmill and measured 2.75 m high and 2.25 m wide. For each trial, we only instructed subjects to “walk while looking down the hallway” so that subjects could naturally adapt to the visual information provided.

In different 3 min conditions, subjects walked with and without a series of continuous mediolateral visual perturbations consisting of a sum of sinusoids applied such that the fore-ground translated at full amplitude while the end of the hall remained nearly stationary. Perturbations were thus designed to elicit balance corrections and not heading corrections on the treadmill. For our primary conditions, we prescribed pairs of driving frequencies for each of five different perturbations according to

(1) P(t)=Asin(f2πt)+Asin(3.1f2πt)

where A prescribed an amplitude of 0.326 m and f was defined as 0.05, 0.1, 0.2, 0.3, or 0.4 Hz. By prescribing the second sinusoidal frequency as a scalar multiple of the first, we preserved peak lateral motion of 0.65 m and the spatial dynamics of each perturbation across conditions (Fig. 1B). We opted for a sum of sinusoids and selected 3.1 as the scalar multiple to promote signal complexity that would be difficult for subjects to anticipate. In addition, the base frequency range encompasses those used in earlier visual perturbation studies, and the scalar multiple extends this range to frequencies faster than stride frequency to test our hypotheses. These perturbations are herein described as Pairs 1-5.

Finally, in three other conditions, subjects walked with visual perturbations described by larger combinations of these driving frequencies. Two of these conditions included 5 driving frequencies with amplitudes of 0.19 m at each frequency (Set 1: {*f}*, Set 2: 3.1·{*f}*) and one included all 10 driving frequencies with amplitudes of 0.09 m at each frequency (Set 3) (Fig. 1C). Amplitudes were selected such that peak lateral motion remained at ∼0.65 m. We randomized the order of all conditions for each subject.

### B. Data Collection

A motion capture system (Motion Analysis Corp., Santa Rosa, CA) operating at 100 Hz captured the three dimensional trajectories of markers placed on subjects' sacrum (a surrogate for their center of mass, CoM) and right and left acromion processes and posterior calcanei. We collected and analyzed 3 continuous minutes of data for each condition.

### C. Data Analysis

We low-pass filtered marker trajectories at 16 Hz using a fourth-order, zero-lag Butterworth filter. We then identified the instants of right and left heel-strikes using the peak anterior heel positions relative to the sacral marker using methods described by Zeni et al. [20]. In addition, we averaged the mediolateral trajectories of the right and left acromion processes to describe the kinematics of subjects' superior trunk, herein referred to simply as “trunk”. We extracted and analyzed at least 330 consecutive steps from each 3 min condition.

Our primary analyses focused on quantifying both the presence of visuomotor entrainment (Hypothesis 1) and the emergence of balance deficits in response to visual perturbations (Hypotheses 2 and 3), the latter evidenced by larger ML trunk motion, increased gait variability, and altered step-to-step temporal persistence. We first used spectral analysis to quantify the intensity of individual visual perturbation frequencies in the ML trajectories of the trunk, sacrum, and right and left heels. Using Matlab (Mathworks, Inc., Natick, MA), we computed the fast Fourier transform (FFT) of the ML marker trajectories during normal and visually perturbed walking. We then calculated the peak amplitude of the FFT (i.e., sway intensity) at each driving frequency included in the visual perturbations. By spacing the lines of the FFT by 0.005 Hz, we minimized spectral leakage at the perturbation frequencies and their higher harmonics.

We then quantified gait variability as a marker of balance control using time series of step widths and step lengths constructed from the distances between the right and left heel markers across successive steps. Specifically, we defined step widths as the ML distance between heel marker positions averaged during midstance prior to heel-rise (i.e., 12-25% stride) [10, 21]. We defined step lengths as the anterior-posterior distance between heel marker positions at 20% of each stride plus the treadmill belt's translation since the previous step [11]. Foot placement variability was characterized as the standard deviations of step width and step length over each trial duration. We then quantified changes in peak-to-peak ML trunk motion elicited by visual perturbations, which we refer to as postural disturbances. Specifically, we calculated the mean and standard deviations of ML trunk translation and of that relative to the pelvis (i.e., trunk “sway”).

Finally, we investigated step-to-step correlations in foot placement and trunk kinematics by calculating the temporal persistence of each outcome measure from their respective time series using detrended fluctuation analysis (DFA) [22, 23]. Specifically, we calculated the root mean square (RMS) of linearly detrended residuals over a range of steps between 4 and N/4, where N described the total number of steps available. Finally, we computed the scaling exponent, *α,* of the relation between the RMS residual of detrended step width and the number of steps detrended. We interpreted *α* as follows: *α* = 0.5 indicates uncorrelated white noise, *α* < 0.5 indicates that deviations in one direction are likely to be followed by deviations in the opposite direction (i.e., anti-persistent), and *α* > 0.5 indicates that deviations in one direction are likely to be followed by deviations in the same direction (i.e., persistent). For example, we previously found that step width during normal, unperturbed walking is temporally persistent, implying that a wide left step is statistically more likely to be followed by a wide right step [10].

### D. Statistical Analysis

Shapiro-Wilk tests first confirmed that all outcome measures across all conditions were normally distributed except for the temporal persistence of step length during normal walking. A two-way repeated measures analysis of variance (rmANOVA) then tested for significant main effects of and interactions between condition and anatomical location (trunk, sacrum, heel) on peak spectral values. A second rmANOVA tested for significant main effects of condition on trunk sway, gait variability, and DFA scaling exponents. When a significant main effect was found, we performed post-hoc pairwise comparisons using Tukey's HSD and a significance level of p<0.05. We also performed one-sample t-tests to evaluate the presence of temporal persistence or anti-persistence in DFA scaling exponents (i.e., *α* ≠0.5).

## III. Results

### A. Evidence for visuomotor entrainment during walking

**Fig. 2:**
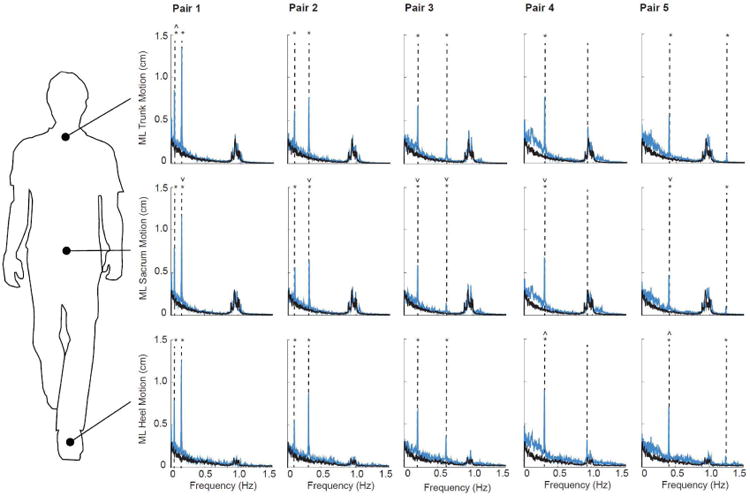
Group average spectrum of trunk, sacrum, and heel mediolateral motion for visually perturbed walking (Pairs 1-5, blue lines) compared to normal, unperturbed walking (black lines). Vertical dashed lines indicate the driving frequencies included in each visual perturbation: Pair 1 (0.05 and 0.155 Hz), Pair 2 (0.10 and 0.31 Hz), Pair 3 (0.20 and 0.62 Hz), Pair 4 (0.30 and 0.93 Hz), and Pair 5 (0.40 and 1.24 Hz). Asterisks (*) indicate significant difference between normal and visually perturbed walking at a given driving frequency (p<0.05). Carets (^˄^) and inverse Carets (^˅^) indicate significantly larger and smaller responses compared to both other anatomical positions at a given driving frequency, respectively (p<0.05).

**Fig. 3:**
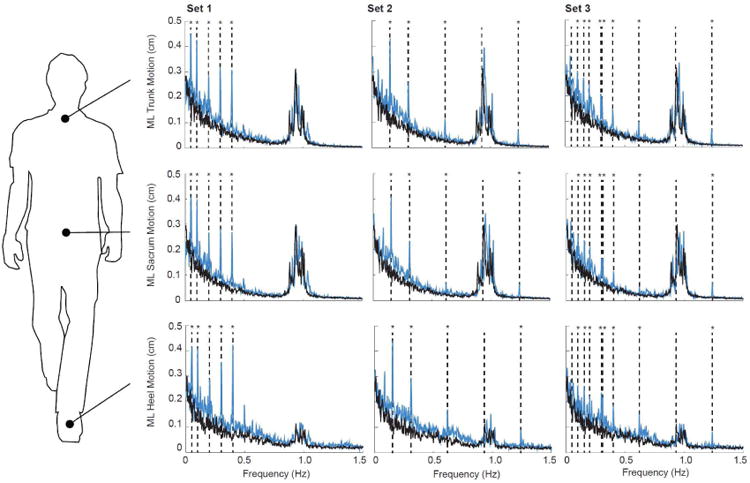
Group average spectrum of trunk, sacrum, and heel mediolateral motion for visually perturbed walking (Sets 1-3, blue lines) compared to normal, unperturbed walking (black lines). Vertical dashed lines indicate the driving frequencies included in each visual perturbations: Set 1 (0.05, 0.10, 0.20, 0.30, and 0.40 Hz), Set 2 (0.155, 0.31, 0.62, 0.93, and 1.24 Hz), and Set 3 (0.05, 0.10, 0.155, 0.20, 0.30, 0.31, 0.40, 0.62, 0.93, and 1.24 Hz). Asterisks (*) indicate significant difference between normal and visually perturbed walking at a given driving frequency (p<0.05).

During normal, unperturbed walking, ML kinematics of the trunk, sacrum, and heels were dominated by signal intensities near subjects' stride frequency (0.96 ± 0.04 Hz) (Fig. 2). In contrast, these kinematics were acutely and significantly affected by visual perturbations (condition main effects, p's < 0.01) (Fig. 2). For our primary conditions (i.e., Pairs 1-5), the intensity of ML motion at all but the second fastest driving frequency increased by at least three-fold and, in some cases, by more than an order of magnitude compared to walking normally (p's<0.01). In addition, significant interactions revealed that subjects' dynamic response to visual perturbations at the majority of driving frequencies differed by anatomical location. Specifically, ML sacrum trajectories generally exhibited significantly smaller responses to visual perturbations than ML trunk or heel trajectories (Fig. 2). ML trunk trajectories exhibited the largest response to the slowest driving frequency (i.e., 0.05 Hz, p<0.01) and ML heel trajectories exhibited the largest response to the slow frequency in Pairs 4 and 5 (i.e., 0.30 and 0.40 Hz, p<0.05 and p=0.02, respectively). Finally, ML kinematics of the trunk, sacrum, and heels exhibited the spectral resolution to resolve up to 8 simultaneously presented driving frequencies (Fig. 3). Indeed, we observed distinct and statistically significant increases in the intensity of ML motion at the individual frequencies comprising Sets 1-3.

### B. Effect of visual perturbations on postural disturbancesand gait variability

**Fig. 4:**
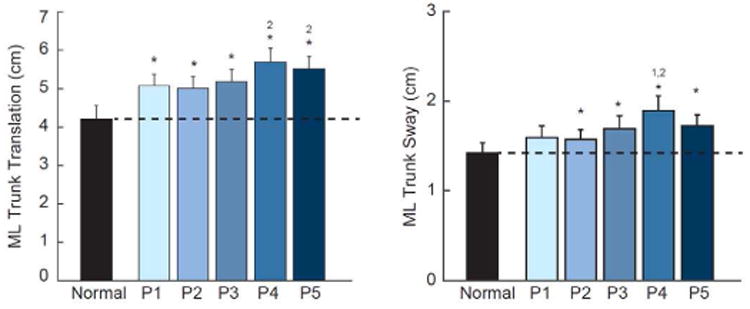
Group average (standard error) measures of postural sway during visually perturbed walking compared to normal, unperturbed walking for our primary conditions. Asterisks (*) indicate significant difference between normal and visually perturbed walking and superscript numbers indicate significant differences between visual perturbations (e.g., ‘2’ refers to Pair 2) (p<0.05).

**Fig. 5:**
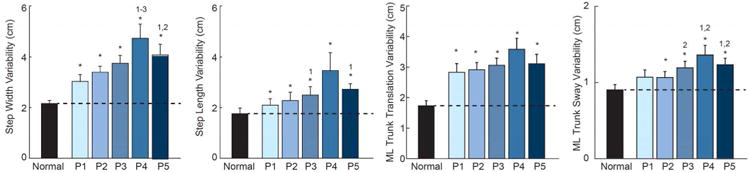
Group mean (standard error) measures of foot placement and postural sway variability during visually perturbed walking compared to normal, unperturbed walking for our primary conditions. Asterisks (*) indicate significant difference between normal and visually perturbed walking and superscript numbers indicate significant differences between visual perturbations (p<0.05).

Compared to normal walking, ML trunk translation and sway (Fig. 4), and their respective step-to-step variabilities (Fig. 5), increased significantly in response to visual perturbations at all driving frequencies (p<0.01). However, compared to that elicited by the other perturbations, these postural disturbances were up to three times larger for Pair 4, which also corresponded to the perturbation nearest subjects' average stride frequency. Step width variability and step length variability also increased substantially from normal walking in response to visual perturbations at all driving frequencies, with the largest changes also observed for Pair 4 (p<0.01) (Fig. 5). Most notably, compared to normal walking, step width variability increased by 144% in response to the perturbation in Pair 4.

### C. Effect of visual perturbations on temporal persistence

Time series of step width and step length exhibited temporal persistence during normal, unperturbed walking (width: *α =* 0.67 ± 0.09, p<0.01; length: *α =* 0.58 ± 0.08, p=0.02) (Fig. 5). Except for Pair 4, visual perturbations significantly decorrelated both step width and step length. Although not significantly different from normal walking (p=0.12), only Pair 4 tended to strengthen the temporal persistence of step width (α = 0.77 ± 0.16), which was 36 and 49% greater than that of perturbations with the nearest driving frequencies (i.e., Pairs 3 and 5, p's≤0.01). In contrast, time series of peak-to-peak trunk translation exhibited anti-persistence during normal walking and during visually perturbed walking for Pairs 2, 3, and 5 (α < 0.5, p's<0.01) (Fig. 5). The driving frequencies in Pairs 1 and 4 uniquely decorrelated step-to-step changes in trunk translation such that these were not significantly different from uncorrelated white noise (Pair 1: *α =* 0.44±0.14, p=0.17; Pair 4: *α =* 0.43 ± 0.15, p=0.15). Pairs 1 and 4 simultaneously elicited temporal persistence in ML trunk sway (p's<0.02) that was not present during normal walking nor for any other perturbation.

## IV. Discussion

In this study, we sought to systematically investigate the prevalence and resolution of visuomotor entrainment in human walking and its role in the emergence of balance deficits due to visual perturbations. Our findings generally supported each of our hypotheses. First, as hypothesized, the spectrum of mediolateral motion of the trunk, sacrum, and heels exhibited naturally emerging and robust entrainment to visual perturbations across a broad range of driving frequencies of perceived mediolateral motion. Also as hypothesized, we found evidence that the effects of visual perturbations on walking balance were frequency-dependent, governed by their proximity to stride frequency. As we elaborate below, visuomotor entrainment may allude to mechanisms by which visual feedback is utilized in motor planning and execution to control walking balance, thereby improving our understanding of the emergence of balance deficits with visual perturbations

Mediolateral motion of the trunk, sacrum, and heels was highly sensitive to visual perturbations. Distinct peaks at the majority of visual perturbation frequencies emerged in the spectrum of mediolateral motion, which may arise through a complex series of sensorimotor pathways. We suspect that these dynamic responses were driven foremost by visual kinesthesis [13], wherein head and trunk movements were regulated via postural control to unify visual with vestibular feedback. This interpretation is consistent with studies on the postural control of standing, which speculate that the integration of visual information minimizes errors between the visual perception of motion and actual motion of the head and trunk [12]. Furthermore, the experiments of Pozzo et al. [24] and others [14, 25, 26] emphasized the importance of head and trunk stabilization during walking. Accordingly, we presume that acute changes in the spectrum of mediolateral heel trajectories arise in principal as a mechanical consequence of postural adjustments during visually perturbed walking. It is unlikely that leg muscle adjustments alone could explain the observed spectral resolution of heel trajectories, especially for the larger combinations of driving frequencies in Sets 1-3.

Visuomotor entrainment, presumably of trunk motion, subsequently elicited frequency-dependent changes in postural disturbances, gait variability, and temporal persistence. As hypothesized, despite eliciting strong visuomotor entrainment of trunk motion, slower driving frequencies that allowed for the planning and execution of corrective actions via foot placement over several steps least affected metrics of balance. In contrast, for faster visual perturbations, visuomotor entrainment became progressively more linked with increases in gait variability. These more variable kinematics suggest that postural disturbances during visually perturbed walking became more difficult to accommodate via foot placement with increasing perturbation frequency. However, compared to the perturbation eliciting the largest increase in postural disturbances and kinematic variability (i.e., Pair 4), these measures tended to decrease for Pair 5 which included a driving frequency faster than subjects' stride frequency. Conceptually, because of the speed of this sinusoidal perturbation, any brief sense of imbalance in a given direction would, based on visual feedback alone, appear to have already been corrected before the next step begins. Thus, as hypothesized, our observations for Pair 5 may reflect a form of neuromechanical filtering, wherein perturbations faster than stride frequency and the normal mediolateral oscillation of the body are effectively mitigated in walking balance control.

The interaction between visual perturbation frequency and gait variability also suggests that, as hypothesized, there is a frequency band of perceived mediolateral motion for which the control of balance is most compromised. In this study, the largest changes in postural disturbances and gait variability emerged in response to the perturbation in Pair 4, containing two driving frequencies - 0.30 Hz and 0.93 Hz. As a consequence of our design, Pair 2 contained a neighboring driving frequency of 0.31 Hz and, compared to Pair 4, elicited substantially smaller changes in postural disturbances and foot placement variability. Thus, we deduce that balance metrics were most sensitive to the 0.93 Hz driving frequency in Pair 4, which also represented the driving frequency in this study nearest to subjects' average stride frequency. Unlike slower perturbations, any driving frequency between one half stride frequency and stride frequency would permit on average only a single step to plan and execute an adjustment in foot placement in response to a sense of imbalance in a given direction. Moreover, with closer proximity of the driving frequency to stride frequency, progressively less time would be available to sample a perceived change in mediolateral trunk motion and plan an appropriate adjustment in foot placement for this step. As we elaborate in more detail below, our findings may suggest that adjustments in foot placement executed in response to the 0.93 Hz driving frequency were decoupled from the postural disturbances ultimately elicited by the perturbation, thereby explaining the marked increase in kinematic variability for Pair 4. Moreover, these findings corroborate stride frequency as an important determinant of the temporal resolution of foot placement control in maintaining balance during walking. Indeed, models relevant to the neural control of walking predict as a prerequisite planning two steps (i.e., one stride) ahead to maintain balance in nearly all situations [27].

Based on the above interpretation, we would expect a fundamentally different pattern of step-to-step adjustments for posture versus foot placement to emerge for Pair 4 compared to other visual perturbations. Indeed, our DFA findings revealed changes in step-to-step correlations unique to Pair 4. During unperturbed walking, step width exhibited temporal persistence, such that a wide left step was more likely to be followed by a wide right step and vice versa. In a prior study [10], we described this step to step dependence of step width as an important characteristic of normal walking, acting to delimit ML fluctuations in CoM motion. In contrast, for all but Pair 4, visual perturbations eliminated this step-to-step dependence and decorrelated step-to-step changes in step width. These changes were accompanied by stronger temporal anti-persistence of peak-to-peak trunk translation.

These findings imply that for most conditions, pseudorandom visual perturbations infiltrated the planning and execution of foot placement as subjects sought to rapidly correct postural disturbances on a step by step basis. In contrast, unlike subjects' response to any other perturbation, we observed stronger temporal persistence of step width and a decorrelation of trunk translation for Pair 4. These unique changes in the step to step control of foot placement and trunk motion are consistent with our explanation for the increased kinematic variability also observed for Pair 4. Specifically, with insufficient time to plan and execute adjustments in response to postural disturbances, foot placement for this condition alone remained highly dependent on that of the prior step. This step-to-step dependence would be expected if foot placement were decoupled from the postural disturbances elicited by Pair 4 as described, requiring systematic corrections over consecutive steps. We suspect that this behavior also explains the simultaneous decorrelation of trunk translation, implying that postural disturbances were not well corrected from one step to the next.

One of our most surprising findings was the extraordinary spectral resolution in which the frequency content of visual information directly mapped onto ML motion. Prior studies have demonstrated the importance of head and trunk stabilization via postural control in walking [14, 24-26]. Our findings underscore these earlier results and reveal a precision in visuomotor control capable of entrainment to up to 8 of 10 simultaneously presented driving frequencies. This shows that visuomotor pathways are exceptionally sensitive to the perception of mediolateral motion (i.e., visual kinesthesis) and orchestrate task-specific kinematic adjustments to unify sensory feedback information. It is also interesting to note that these responses emerged without the appearance of higher harmonics of the driving frequencies, even though the neu-romechanics of gait are considered to be nonlinear [16]. Future studies are needed to explore the extent to which this spectral resolution is preserved or compromised with sensory-induced balance deficits due to aging, injury, or disease. In addition, these empirical data provide opportunities to evaluate neuromechanical models of walking to further explore the interactions between perceived mediolateral motion and acute adjustments in trunk and foot placement kinematics. Finally, electromyographic measurements would be important future contributions to identify the origin of postural adjustments, and to better differentiate passive mechanics from active control in trunk motion and foot placement during walking.

The fundamental insights gained from this study in healthy young adults allude to several possible explanations for our previous observation; compared to young adults, old adults were found to exhibit a remarkable increase in their sensitivity to visual perturbations during walking [10, 11]. We also reported considerably stronger entrainment in old than young adults to the two driving frequencies used in those studies (i.e., 0.14 and 0.44 Hz) [10]. In the present study, we describe visuomotor entrainment in young adults as their attempt to unify visual with vestibular feedback. This explanation may suggest that otherwise healthy old adults retain the capacity to identify such sensory inconsistencies, but execute postural adjustments that are enhanced compared to young adults. This would further support our prior interpretation [10] and that of others [7] that old adults' sensitivity to visual perturbations reflects an increase in visual feedback gain. In addition, we previously found that ML balance was significantly more compromised in old than young adults by visual perturbations. Thus, coordination between postural adjustments and foot placement control may be especially compromised by aging, likely precipitated by reductions in old adults' somatosensory function. Indeed, we have suspected that impaired peripheral sensation may be one factor contributing to the reliance on visual information to maintain balance in old adults. Moreover, here we show that balance is most negatively affected when adjustments in foot placement are decoupled from the postural disturbances elicited by visual perturbations.

There are several important limitations of this study. First, we studied average responses during 3-min exposure to visual perturbations, and the propensity for visuomotor adaptation and thus time-dependent changes in our outcome measures is not well understood. In addition, our findings may be specific to continuous mediolateral perturbations and may not reflect adjustments in trunk motion and foot placement in response to a more discrete sense of imbalance. Further, future work may consider assessing the phasing between visual perturbations, postural disturbances, and foot placement, which we were unable to evaluate. We also used metrics of balance derived solely from kinematic measures which may not fully capture the complexities of dynamic balance control. Finally, we interpret the observed changes in ML trunk, sacrum, and heel marker trajectories to be evidence of visual information used in a feedback, rather than feedforward manner. We designed our visual perturbations to promote signal complexity that would be difficult for subjects to predict. We cannot fully exclude the possibility that subjects anticipated the perturbations and adjusted their kinematics according to feedforward control. However, this would seem highly unlikely, especially for the larger combinations of perturbation driving frequencies.

## V. Conclusion

We conclude that visuomotor entrainment is a robust and naturally emerging phenomenon during human walking, involving adjustments in postural control at frequencies directly present in the available visual information. Metrics of walking balance exhibit a complex, frequency-dependent response to visual information; foot placement and trunk motion were particularly disrupted when visual perturbations included information at frequencies near subjects' average stride frequency. Finally, our findings allude to a remarkable level of sensitivity governing visuomotor entrainment in walking. These findings provide mechanistic insight into how walking balance can be directly disrupted by mediolateral perturbations of optical flow. The technical approach and data may provide a baseline for identifying the emergence of balance deficits due to age, injury, or disease.

## Figures and Tables

**Fig. 6:**
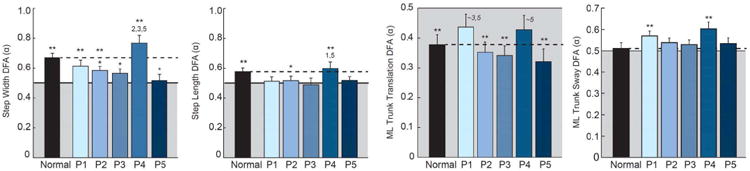
Group mean (standard error) measures of foot placement and postural sway temporal persistence during visually perturbed walking compared to normal, unperturbed walking for our primary conditions. Horizontal black lines represent DFA scaling exponents corresponding to uncorrelated white noise (i.e., *α* = 0.5), and gray region represents those corresponding to temporal anti-persistence (i.e., *α* < 0.5). Single asterisks (*) indicate significant difference between normal and visually perturbed walking and double asterisks (**) indicate significantly different from uncorrelated white noise (p<0.05). Superscript numbers indicate significant differences between visual perturbations (p<0.05).
